# What is the preferred concentration of ethanolamine oleate for sclerotherapy of oral vascular anomalies?

**DOI:** 10.4317/medoral.23448

**Published:** 2020-05-10

**Authors:** Camila de Nazaré Alves de Oliveira Kato, Michel Campos Ribeiro, Mauro Henrique Nogueira Guimarães de Abreu, Soraya de Mattos Camargo Grossmann, Lucas Guimarães Abreu, Patrícia Carlos Caldeira, Ricardo Alves Mesquita

**Affiliations:** 1 DDS, MSc, PhD student. Dept. of Oral Surgery and Pathology, School of Dentistry, Universidade Federal de Minas Gerais, Brazil; 2 DDS, PhD, Oral and Maxillofacial Surgeon. Dept. of Oral and Maxillofacial Surgery, Hospital Márcio Cunha, Brazil; 3 DDS, PhD, Professor. Dept. of Social and Preventive Dentistry, School of Dentistry, Universidade Federal de Minas Gerais, Belo Horizonte, MG, Brazil; 4 DDS, PhD, Professor. Dept. of Oral Pathology, Pontifical Catholic University of Minas Gerais (PUC Minas), Brazil; 5 DDS, PhD, Professor. Dept. of Pediatric Dentistry and Orthodontics, School of Dentistry, Universidade Federal de Minas Gerais, Brazil; 6 DDS, PhD, Professor. Dept. of Oral Surgery and Pathology, School of Dentistry, Universidade Federal de Minas Gerais, Brazil

## Abstract

**Background:**

This study compared three different concentrations of EO (1.25%, 2.5% and 5%) for the treatment of oral vascular anomalies (OVAs).

**Material and Methods:**

This was a retrospective comparative analysis of patients with OVAs treated with EO. Anomalies smaller than 20 mm were included. The patients were treated with 1.25% (G1), 2.5% (G2), and 5% (G3) and clinical data were obtained. The number of sessions, the final volume and dose of EO were statistically analyzed to verify effectiveness and safety of the treatment. The different concentrations of EO were compared considering the number of sessions, the final volume and total dose of EO. Analysis of covariance (ANCOVA) was used to evaluate the influence of covariates on the outcomes. A *p*-value < 0.05 was considered significant.

**Results:**

Nineteen women and 11 men with a median age of 54 years were included. The OVAs were most frequent in the lip (n=14) and cheek (n=9). All lesions exhibited complete clinical healing within 28 days. Patients of G3 required fewer sessions than those of G2 (*p*=0.017), a lower final volume compared to the other groups (*p*<0.001), and a lower total dose than G1 (*p*<0.001). Patients of G1 used a lower total dose than G2 (*p*=0.003).

**Conclusions:**

The concentration of 5% EO performed better than 1.25% and 2.5% for sclerotherapy of OVAs measuring up to 20 mm. This preliminary result should be the preferred concentration of EO to provide an effective and safe treatment of OVAs.

** Key words:**Ethanolamine oleate, sclerotherapy, vascular malformations, hemangioma, oral mucosa.

## Introduction

Sclerotherapy is an important and well-recognized treatment modality for oral vascular anomalies (OVAs). Effectiveness is observed in more than 70% of the cases treated with sclerosing agents (1). The intralesional injection of the sclerosing agent causes irritation in the endothelium followed by an inflammatory response (2,3) whose outcomes include fibrosis of the vessel wall or vein obliteration. The sclerosing agent also diffuses rapidly through the vessel wall and produces extravascular inflammation (4). Sclerotherapy has been largely used for the management of esophageal varix (3,5), oral hemangioma, vascular malformations, and varices (6-9).

*Pi*ngyangmycin, OK-432, doxycycline, sodium tetradecyl sulfate, polidocanol, hypertonic saline, absolute alcohol, bleomycin, and ethanolamine oleate (EO) are available for sclerotherapy of OVAs (6,7,10,11). Ethanolamine oleate is an unsaturated fatty acid, known for its lower toxic effects compared to other sclerosing agents (2,7,12). However, the optimal concentration of EO to treat oral OVAs has not been determined. Previous studies have shown a good response when a concentration of 1.25%, 2.5% or 5% was used for the treatment of OVAs, but there were differences in the number of sessions and the final volume of EO necessary to achieve complete resolution (2,6,7,9).

Establishing the optimal concentration of EO for sclerotherapy of OVAs is important to provide an effective, safe and timeless treatment, but also to prevent or reduce side effects such as rash, edema, pain, bleeding, ulceration, or necrosis. Therefore, the aim of this preliminary study was to compare the effectiveness and safety of sclerotherapy with EO for the treatment of OVAs using concentrations of 1.25%, 2.5% and 5%. Our hypothesis is that 5% EO is more effective and safer, requiring fewer treatment sessions and a lower final volume and total dose.

## Material and Methods

- Study design and sample

A retrospective and comparative study was carried out using the records of patients diagnosed with OVAs, who were treated by a single operator (R.A.M.) of the service of Oral and Maxillofacial Pathology Service, School of Dentistry, Federal University of Minas Gerais, over the past 10 years.

The diagnosis of OVAs was established based on clinical criteria and on the history of the anomaly according to Mulliken and Glowacki (13) using the latest classification for vascular anomalies approved by the International Society for the Study of Vascular Anomalies workshop (14). Functional or esthetic criteria were used for treatment indication.

All OVAs were treated with EO (Ethamolin™, ZEST, Rio de Janeiro, RJ, Brazil). Topical anesthetic gel (Benzocaine 20%, Nova DFL, Rio de Janeiro, RJ, Brazil) was applied over the OVA for 60 seconds prior to EO injection. A short insulin needle and a 1 CC syringe (BD, Juiz de Fora, MG, Brazil) were used for the application of EO. Blood was aspirated to determine whether the needle was inserted into the vascular lumen. After injection, pressure was applied to the OVA with gauze for about 3 minutes to prevent reflux of EO. The patients returned within 7 days for clinical follow-up. For patients who required more than one application to achieve complete clinical resolution, an interval of 14 days between each application was followed.

For the current retrospective analysis, consecutively treated cases of OVAs were selected and only OVAs smaller than 20 mm were included to avoid sample variations. The patients were divided into three groups according to the concentration of EO applied. Group 1 (G1) consisted of patients treated with EO diluted 1:4 in sterile water, at a final concentration of 1.25%. Group 2 (G2) was treated with EO diluted 1:1 in distilled water, at a final concentration of 2.5%. Group 3 (G3) received undiluted EO at a final concentration of 5%. For G1 and G2, EO was prepared following the protocol described by Johann *et al*. (6). For G3, the drug administration protocol was based on Costa *et al*. (7).

- Variables

The following data were collected from the patient charts: sex, age, and skin color of the patient; clinical diagnosis, site, and size of the OVAs; side effects of treatment (rash, edema, pain, ulceration, bleeding, and necrosis); clinical healing, number of sessions performed, and final volume and total dose of EO used. Pain and edema scores were not available in the participants’ records. Therefore, the retrospective design of the study did not permit the retrieval and evaluation of data on pain and edema.

- Statistical analysis

Data analysis was carried out using the Statistical Package for the Social Sciences 17.0 (SPSS, Inc., Chicago, IL). The quantitative variables age, OVA size, number of sessions, final volume, and total dose were tested regarding normality by means of the Shapiro-Wilk test and a non-normal distribution was confirmed. Therefore, two or more groups were compared using the Kruskal-Wallis and Mann-Whitney tests for quantitative data and Fisher’s exact test for categorical variables. Fisher’s exact test was used to evaluate the differences in categorical variables. The different concentrations of EO were compared considering the number of sessions and the final volume and total dose of EO. Analysis of covariance (ANCOVA) was used to evaluate the influence of covariates on the outcomes. A *p-value* < 0.05 was considered significant.

## Results

Records of 30 patients (19 women and 11 men) were included in this study. The age of the patients ranged from 11 to 72 years (median: 54 years). Sixteen patients were white-skinned and 11 were non-white. Among the 30 OVAs, 10 were classified as hemangiomas, 10 as vascular malformation, and 10 as varices. The most frequently affected site was the lip (n=14), including the lower (n=11) and upper lip (n=3), followed by the cheek (n=9), palate (n=3), tongue (n=2), gingiva (n=1), and alveolar mucosa (n=1). All lesions had healed completely within 28 days. Local pain and edema were reported by all patients up to 72 hours after the administration of EO. As local pain and edema were not quantified, they were not considered in the statistical analysis. No other side effects were reported. Table 1 shows the comparison of participants’ age, clinical diagnosis of OVAs, and OVA size.

There was no difference in sex among groups (Fisher’s exact test, *p*=0.51). The groups differed in terms of age (Kruskal-Wallis test, *p*=0.043) and clinical diagnosis (Fishers exact test, *p*=0.004). Patients of G1 were significantly younger than those of G3 (Mann-Whitney test, *p*=0.016). Significant differences in the clinical diagnosis were also observed between G1 and G3 (Fisher’s exact test, *p*=0.001), with a predominance of hemangioma cases in G1 (n=7) and of varices in G3 (n=6). There was no significant difference in OVA size between groups (Kruskal-Wallis test, *p*=0.15).

The Kruskal-Wallis test showed significant differences among groups for the variables number of sessions (*p*=0.047), final volume (*p*<0.001), and total EO dose (*p*<0.001) (Table 2). Comparisons between groups by the Mann-Whitney test revealed a significant difference in the number of sessions between G3 and G2 (*p*=0.017). The final volume of EO was lower in G3 compared to G1 (*p*<0.001) and G2 (*p*<0.001). The total EO dose was higher in G2 than in G1 (*p*=0.003) and G3 (*p*<0.001). After ANCOVA, the differences in the total dose (*p*=0.036) between G1 and G2 and in final volume between G1 and G3 (*p*=0.016) remained statistically significant, regardless of participant age, clinical diagnosis, and OVA size (Table 3). ANCOVA was not necessary for the comparison between G2 and G3 because these two groups were similar in terms of participant age (*p*=0.478), clinical diagnosis (*p*=0.25), and OVA size (*p*=0.87).

**Table 1:**
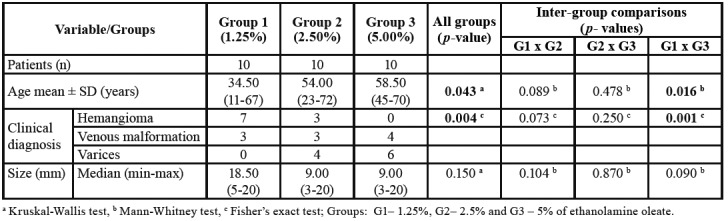
Clinical data of the patients treated with ethanolamine oleate for oral vascular anomalies.

**Table 2:**
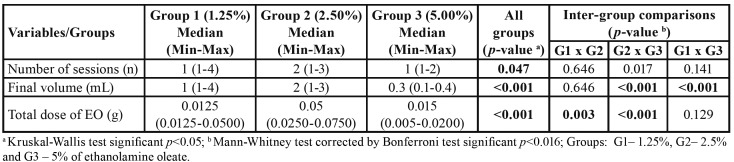
Comparison of the different concentrations of ethanolamine oleate to treat oral vascular anomalies.

**Table 3:**
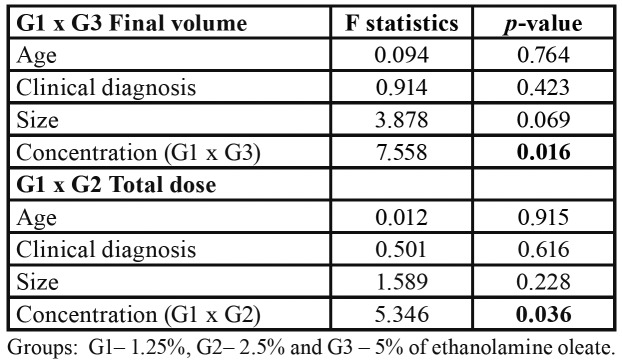
Analysis of covariance (ANCOVA) for final volume and total dose of ethanolamine oleate to treat oral vascular anomalies.

## Discussion

This preliminary study compared three different EO concentrations for the treatment of OVAs. The hypothesis that 5% EO (G3) requires a lower final volume and total dose and fewer sessions was confirmed. Using 5% EO, only one or two sessions were necessary for complete clinical resolution of the lesion, whereas at concentrations of 1.25% and 2.5%, the number of sessions ranged from one to four. A reduction in the number of sessions associated with the use of 5% EO was also observed by Costa *et al*. (7) and Fernandes *et al*. (9). The use of 1.25% and 2.5% EO may require one to 10 sessions for the complete clinical resolution of OVAs (2,6,15).

A recent systematic review on sclerosing agents has shown that pingyangmycin, absolute ethanol, OK-432, EO, bleomycin, polidocanol, doxycycline, and sodium tetradecyl sulfate appear to be effective, although potential complications associated with each agent should be considered when selecting a drug (1). Pain/burning upon injection, temporary scars, psychological issues, rash, ulcerations, bleeding, and the risk of local necrosis are complications of sclerotherapy (1,8,16). For EO, these adverse effects are mainly related to the dose applied, and a total dose of 0.4 ml/kg has been considered the maximum safe dosage of EO (13,17). The size of OVAs is also an important factor for sclerotherapy mainly because of the possible toxic effect of EO. The absence of the other side effects was expected because of the pre-defined limitation of OVA size, smaller than 20 mm, which required a lower dose of EO ranging from 0.005 g to 0.075 g, and even lower dose when EO was used at a concentration of 5%. Sclerotherapy of anomalies larger than 20 mm is likely to result in more adverse effects (52.9%) such as superficial necrosis, and thus requires careful monitoring (18).

There was no difference in sex among the participants, but patients of G1 were younger than those of G3. Patient age may have influenced the results, as there was a difference between groups. A reduced inflammatory response is observed in the elderly due to the physiological aging process (19-21). The OVAs were larger in G1 than in G2 and G3. After ANCOVA, the differences in the total dose between G1 and G2 and in the final volume between G1 and G3 were attenuated, but remained significant regardless of the participants’ age, clinical diagnosis of OVA, and OVA size. The size of the lesion and the clinical diagnosis are important variables for evaluation of the clinical response. Larger lesions may require more sessions and consequently a higher volume and final dose.

Numerous techniques such as surgery, embolization, laser therapy, systemic corticosteroids, cryotherapy, interferon α and radiation therapy have been recognized as treatments of OVAs (8,22-25). Deciding upon which technique will offer the best results depends on the size, location and hemodynamics of the lesion, the degree of invasion into anatomic structures, and the age of patients (8,26). The sclerosis technique consists of direct percutaneous puncture and is an easy, simple, fast and low-cost technique that can be carried out in an outpatient setting with low morbidity and well tolerated by the affected individual. The clinician’s experience also contributes to successful sclerotherapy (9). For several sclerosing agents, sclerotherapy is effective in more than 70% of cases of cervicofacial anomalies, while EO has a success rate higher than 88% (1). In our study, the inclusion of OVA cases smaller than 20 mm may explain the success rate in all cases of the present sample treated with three different concentrations of EO.

With respect to the technique, we used a topical anesthetic prior to EO injection. In addition, distilled water was used to dilute EO, as previously reported (2,15). However, other studies have recommended the intravascular application of EO diluted in 2% lidocaine and epinephrine (1:100,000), as well as anesthetic infiltration with vasoconstrictor around the lesion (8,9). The objective of topical application of the anesthetic is to avoid the possible interaction between both drugs. Moreover, the use of infiltrative anesthesia may hinder visualization of the lesion and interfere with the technique of EO injection.

This study has some limitations. The retrospective study design with data extraction from medical records has disadvantages, although the treatment was performed by the same service. The pain and edema scores of individuals treated with the three different concentrations of EO could have been relevant information for clinical practice. However, information on pain or edema scores was not available in the participants’ records. Therefore, the retrospective design of the study did not permit assessment of these data. Another limitation is the heterogeneity among groups with respect to participant age and clinical diagnosis. Despite the similarity between G2 and G3, there was a difference in age and clinical diagnosis between G1 and G2 and between G1 and G3. We therefore applied ANCOVA to assess differences in the total dose between G1 and G2 and in the final volume between G1 and G3 considering age, clinical diagnosis and size of the lesion as confounders. The difference in final volume between G1 and G3 and in the total dose between G1 and G2 remained significant, regardless of the influence of confounders. Randomized clinical trials are needed in the future to allow a more appropriate evaluation of treatments.

## Conclusions

Despite the cited limitations, our preliminary results demonstrate that 5% EO for the treatment of OVAs smaller than 20 mm resulted in a lower final volume, lower total dose and a tendency towards fewer sessions than 1.25% and 2.5%. This should therefore be the preferred concentration of EO to treat OVAs of 20 mm or less. Further studies, especially randomized clinical trials, are warranted to establish an assertive protocol for OVA sclerotherapy. However, these preliminary results may help clinicians choose the EO concentration for the treatment of OVAs.
